# dPABBs: A Novel *in silico* Approach for Predicting and Designing Anti-biofilm Peptides

**DOI:** 10.1038/srep21839

**Published:** 2016-02-25

**Authors:** Arun Sharma, Pooja Gupta, Rakesh Kumar, Anshu Bhardwaj

**Affiliations:** 1 Open Source Drug Discovery (OSDD) Unit, Council of Scientific and Industrial Research (CSIR), New Delhi, India; 2 Academy of Scientific and Innovative Research (AcSIR), CSIR-OSDD Unit, CSIR-HQ, New Delhi, India; 3 Bhaskaracharya College of Applied Sciences, University of Delhi, New Delhi, India

## Abstract

Increasingly, biofilms are being recognised for their causative role in persistent infections (like cystic fibrosis, otitis media, diabetic foot ulcers) and nosocomial diseases (biofilm-infected vascular catheters, implants and prosthetics). Given the clinical relevance of biofilms and their recalcitrance to conventional antibiotics, it is imperative that alternative therapeutics are proactively sought. We have developed dPABBs, a web server that facilitates the prediction and design of anti-biofilm peptides. The six SVM and Weka models implemented on dPABBs were observed to identify anti-biofilm peptides on the basis of their whole amino acid composition, selected residue features and the positional preference of the residues (maximum accuracy, sensitivity, specificity and MCC of 95.24%, 92.50%, 97.73% and 0.91, respectively, on the training datasets). On the N-terminus, it was seen that either of the cationic polar residues, R and K, is present at all five positions in case of the anti-biofilm peptides, whereas in the QS peptides, the uncharged polar residue S is preponderant at the first (also anionic polar residues D, E), third and fifth positions. Positive predictions were also obtained for 29 FDA-approved peptide drugs and ten antimicrobial peptides in clinical development, indicating at their possible repurposing for anti-biofilm therapy. dPABBs is freely accessible on: http://ab-openlab.csir.res.in/abp/antibiofilm/.

Microbial antibiotic products and antimicrobial resistance (AMR) are believed to have similarly timed evolutionary origins. Metagenomic analyses (of 30,000-year-old permafrost samples, for instance) have revealed an extensive dissemination of resistance elements across ecosystems^1^. Fleming’s serendipitous discovery of penicillin was our first glimpse into this ancient, microbial world of chemical warfare. For decades since, antibiotics have made possible the edifice of modern medicine, but the selective pressure exerted by their injudicious consumption worldwide has led to the emergence of vengefully resistant bacteria^2^. Antibacterial resistance (ABR) has become a matter of grave concern as an increasing number of clinically relevant pathogens are developing resistance to multiple drugs that include some of the most potent killers available in our antibiotic arsenal. Examples include carbapenem-resistant enterobacteriaceae, pan-resistant *Acinetobacter*, vancomycin-resistant *Enterococcus*, methicillin and vancomycin-resistant *Staphylococcus aureus,* and multi-drug-resistant *Mycobacterium tuberculosis* (estimated to cost the world $16.7 trillion by 2050 3)^4^,^5^. The loss of effective antibiotics will have far-reaching implications; the ability to treat secondary infections is crucial in vulnerable, immunocompromised individuals suffering from severe diabetes or undergoing chemotherapy for cancer, dialysis for renal failure, organ transplantation and surgical procedures. The crisis is aggravated by the dearth of new drugs in the developmental pipeline, sorely needed to replace those that lose their efficacy against resistant bacteria^6^. Meanwhile, even as we struggle to revitalize antibiotic discovery, our prokaryotic friends can be trusted to never run out of ideas. Biofilms are perhaps the quintessence of this microbial ingenuity.

Biofilms are highly structured, sessile communities characterised by cells adhering to a substratum and embedded in a self-produced matrix of extracellular polymeric substances (EPS, major components include polysaccharides, proteins and extracellular DNA)^7^. This phenotypic adaptation appears early in the fossil record (estimated to be roughly 3.25 billion years old) and is integral for survival in diverse environments^8^. Cells in biofilms differ profoundly from their planktonic counterparts in terms of growth rate and gene transcription^9^. The bacterial cells are present in microcolonies (structural units of the biofilm) that are interspersed with a network of open water channels distributing nutrients and oxygen^10^. Yet, a decreasing gradient of nutrient and oxygen availability exists between the surface and the deeper layers of the biofilm^11^. Consequently, fluoroquinolones and tetracycline can only kill the more metabolically active cells found in the outer layers while a lipopeptide can kill the slow-growing cells present in the oxygen and nutrient-deprived inner layers^12^. Biofilms are ubiquitous in natural and human pathogenic systems (account for >99% of microbial life^13^), are predominantly polymicrobial^14^ and are known to be recalcitrant to environmental stresses like biocidal agents, UV damage, metal toxicity, acid exposure, anaerobic conditions, salinity, desiccation, bacteriophages, host immune responses (phagocytosis)^8^.

In the context of infectious human diseases, the general understanding is that acute infections are caused by planktonic bacterial growth, whereas chronic infections are sustained principally by biofilm formation. It is estimated that 60–70% of all nosocomial infections are due to biofilms present on implanted medical devices. 95% of urinary tract infections are associated with a urinary catheter, 80% of pneumonias with mechanical ventilation, and 87% of bloodstream infections with intravascular devices^15^.

Biofilms promote mutability and drastically enhance horizontal gene transfer of plasmid-borne antibiotic resistance^16^. Their multicellular organisation and spatiotemporal heterogeneity make them 10–1000 times more resistant to conventional antibiotics than their planktonic counterparts^17^. However, cathelicidins (a family of antimicrobial host defense peptides) and related small, cationic synthetic peptides, in concentrations insufficient to kill planktonic bacteria evidently inhibit biofilm formation *in vitro*^18^,^19^,^20^,^21^,^22^. Another similar peptide with no discernible antimicrobial activity could effectively prevent biofilm formation in both gram negative and gram positive bacteria at a concentration well below the minimum inhibitory concentration (MIC)^23^. Thus, it becomes abundantly clear that experimental determination or computational prediction of anti-biofilm activity must take into consideration the inherent differences between the planktonic and biofilm lifestyles and not use planktonic cultures as a proxy measure for biofilm growth.

Presently, there are more than 60 FDA-approved peptide drugs in the market, with at least another 640 in different stages of preclinical development and clinical trials^24^. Antimicrobial peptides (AMPs) have also gained importance as prophylactic and therapeutic agents against drug-resistant bacteria and biofilms^25^,^26^. Like AMPs, peptides with anti-biofilm activity are short (12–50 residues, with 2–9 basic arginine and lysine residues), cationic and amphipathic^18^. Insofar as planktonic bacteria are concerned, numerous databases of AMPs^27^ and tools for the identification and design of such peptides^28^,^29^,^30^ have been developed to date. BaAMPs is the only database of AMPs assessed exclusively for their activity against biofilms^31^. Previously, classification models have been developed for predicting the therapeutic potential of peptides^32^,^33^. To the best of the authors’ knowledge, dPABBs (**d**esign **P**eptides **A**gainst **B**acterial **B**iofilm**s**) is the first web server for predicting biofilm-active peptides and creating mutants thereof with improved activity and optimised physicochemical properties.

Our knowledge-based approach to dataset-selection for predicting anti-biofilm peptides is, admittedly, unconventional. Since the exclusively biofilm-inactive peptides retrieved from the BaAMPs database could not constitute a substantive negative dataset, other alternatives had to be considered. As surmised above, peptides found to be inactive against planktonic bacteria could not have been used for the purpose of building computational models capable of reliably predicting anti-biofilm activity. We therefore reasoned that quorum sensing peptides (QSPs) that exist and function within the biofilm, irrespective of their direct or indirect involvement in enabling the biofilm phenotype, would possess a contrasting set of properties as compared to those peptides which disrupt biofilms. QS peptides with reported anti-biofilm/antimicrobial activity were diligently identified and removed (see Methods for dataset preparation) from the final set used for training the models. Despite their anti-biofilm/antimicrobial activity, these 26 peptides were not included in the positive dataset either. This is because the inclusion would have meant that their analogs too could be predicted as ‘biofilm-active’, some of which may exhibit receptor antagonism (a mechanism of quorum quenching)^34^, an intrinsically species-specific way of interfering with the quorum sensing system of any organism. However, the objective of this study is to evaluate peptides only for their broad-spectrum activity against multiple bacterial species. For this reason, peptides with inconclusive anti-biofilm activity (i.e. peptides that were active against one species but inactive against others) were also excluded from the positive dataset such that it contained only those peptides which were found to disrupt the biofilms of all the species they were tested against.

Quorum sensing (QS) is a sophisticated system of intercellular communication in bacteria that involves the production, detection and response to signalling molecules called autoinducers (AI). At low cell densities, AIs diffuse away, but as the bacterial populations grow, the AIs begin to accumulate in the extracellular milieu and reach the threshold concentration required for detection by their cognate cytoplasmic or membrane-bound receptors, following which the collective gene expression is altered. There are specific autoinducing molecules for intra-species signaling (gram positive bacteria use oligopeptides, gram negative bacteria use acyl homoserine lactones) and a “universal language” (autoinducer-2) for inter-species communication. The QS machinery induces genes that control processes which are beneficial when performed in unison, like bioluminescence, sporulation, competence, antibiotic production, biofilm formation, and virulence factor secretion^35^,^36^. Biofilms and QS are intimately linked as they represent aspects of bacterial community behavior. The term ‘sociomicrobiology’ has been used for investigations into the social facets of microbial life, and the interconnections that exist between them^37^.

In 1998, Greenberg and his colleagues first confirmed the role of the las QS in the formation of biofilms by *Pseudomonas aeruginosa*^38^, although subsequently it became apparent that experimental conditions could influence the degree of this correlation^37^. Since then, studies have shown that the three known QS systems in *P. aeruginosa* are functionally involved in the biofilm lifecycle^39^. It has also been demonstrated that the biosynthesis of EPS directed by QS controls the formation of mature biofilm communities in *Vibrio cholera*^40^.

More importantly, QS inhibitor (RNAIII inhibiting peptide, RIP) was reported to have prevented adhesion and biofilm formation both *in vitro* and *in vivo* in *S. epidermidis*, whose virulence is often associated with its ability to form biofilms^41^. Likewise, RIP also inhibited the TRAP/agr systems in *S. aureus* (both TRAP and agr negative strains are incapable of biofilm formation) and emphatically prevented graft infections of methicillin resistant *S. aureus* in rats^42^. In several streptococci, competence-stimulating peptide (CSP)-mediated QS has been implicated in natural genetic transformation and biofilm formation. In *S. mutans* and *S. intermedius*, for instance, CSP appears to operate in the initial stages of the biofilm mode of growth and the inhabitants of biofilms can incorporate antibiotic resistance and virulence-causing elements with at least 10 times the efficiency seen in their planktonic counterparts^43^. Although there is much to learn about the precise nature of QS’s involvement in biofilm formation, maintenance and dispersal, further elucidation is forthcoming^44^,^45^,^46^. The case for its relevance in biofilms is only strengthened by the recognition of the therapeutic potential of quorum quenching in biofilm infections^34^.

In this study, machine learning tools were used to build six Support Vector Machine (SVM) and Weka-based models trained on 80 biofilm-active AMPs (retrieved from the BaAMPs database, also referred to as anti-biofilm peptides, ABPs henceforth) and 88 QSPs (retrieved from QSPpred^47^, cross referenced with Quorumpeps database^48^). The positive dataset consisted of 90 AMPs (80 in the training set +10 in the independent dataset) found to be active against bacterial biofilms (prevented adhesion to substratum, inhibited formation or destroyed existing biofilms), when assayed either *in vitro* or *in vivo*. For the negative dataset, from a total of 220 unique QSPs, one peptide sequence contained pyrrolysine (O), corresponding entries in Quorumpeps were missing for 30 peptides, 68 peptides had cyclic structures and the 7 amidated linear peptides were RNAIII inhibiting (RIPs), all of which were removed. Out of the remaining 114 non-amidated linear peptides, 26 were identified to have antimicrobial or biofilm-inhibitory activity (EDFs, RIPs, RBPs, bacteriocin-inducing) and were excluded to yield the final 88 peptide sequences used. When run on the independent validation dataset, the best model among these performed with an overall accuracy of 95.24%. FDA-approved peptide drugs as well as antimicrobial peptides in clinical development were also analysed to identify potential repurposing candidates with anti-biofilm properties.

## Results

### Data curation

**Figure 1:**
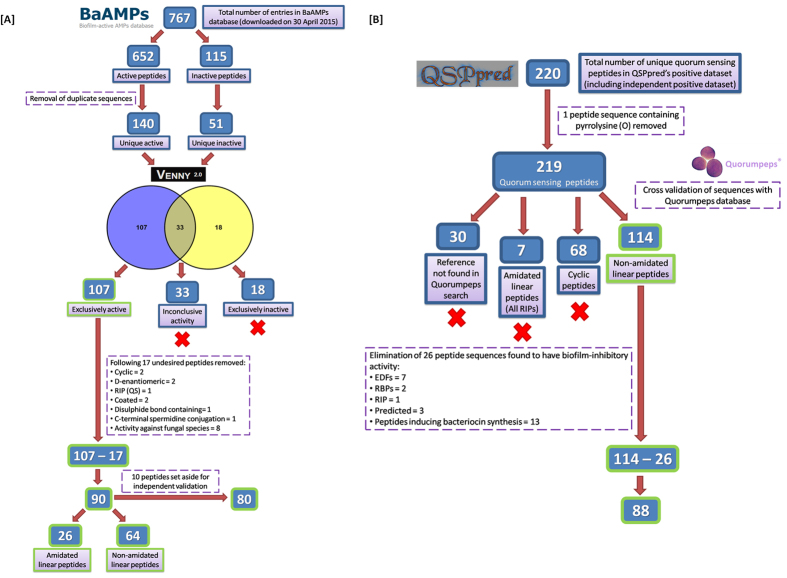
Positive and negative dataset preparation methodology. (**A**) The positive dataset retrieved from the BaAMPs database comprised of 90 ABPs (80 in the training set +10 in the independent dataset). (**B**) The negative dataset retrieved from QSPpred and cross referenced with Quorumpeps database comprised of 88 QSPs (training set).

For machine learning and subsequent model development, the positive (anti-biofilm peptides, ABPs) and negative (quorum sensing peptides, QSPs) datasets were meticulously curated (Fig. 1A,B). For a detailed description of the dataset preparation methodology, please see the Methods section.

**Figure 2:**
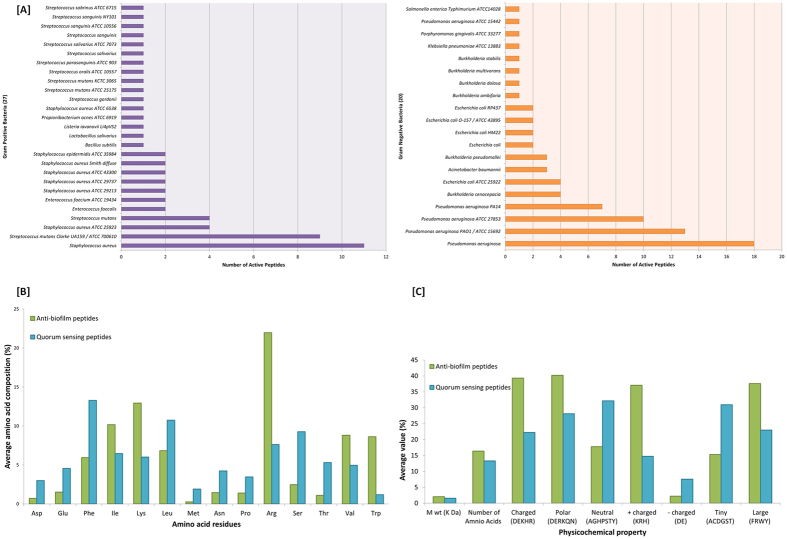
Distribution of amino acids residues and physicochemical analysis of the ABPs and QSPs. (**A**) Frequency distribution of the ABPs tested against the biofilms of gram positive and gram negative bacteria. (**B**) Comparison of the average amino acid percentage composition between the ABPs and QSPs. (**C**) Comparison of the average physicochemical properties (of amino acid residues) between the ABPs and QSPs.

The 80 ABPs used in this study were reported to be active against 27 Gram positive and 20 Gram negative bacteria (Fig. 2A, Listed in Supplementary File [A]).

### Analysis of anti-biofilm and quorum sensing peptides

#### Length of the Peptides

The length of the ABPs used in this study varied from 6 to 36 amino acid residues. Out of main set of 90 sequences, 57 were ≤18 residues in length, with the maximum number of peptides (18) being 12 residues long. The length of the QSPs was found to vary within a similar range of 5 to 30 residues. Out of the main set of 98 sequences, 52 were ≤15 residues in length, with the maximum number of peptides (18) being 5 residues long (Length distribution graphs in Supplementary File [B]).

#### Compositional and Physicochemical analysis

Calculation of the amino acid percentage composition for ABPs and QSPs indicated significantly higher representation of charged residues (D, E, K, H, R) in the ABPs. Furthermore, positively charged residues (K, H, R) were more numerous in the ABPs while the QSPs had more negatively charged residues (D, E). Additionally, the number of polar amino acids was also considerably higher in ABPs whereas QSPs were richer in neutral amino acids. It was also observed that the number of tiny amino acids (A, C, D, G, S, T) was relatively higher in QSPs whereas there were more large amino acids (F, R, W, Y) in the ABPs (Fig. 2B,C)^49^. To quantify the hydropathicity of these peptides, their Grand Average of Hydropathicity (GRAVY) values were estimated^50^. For the ABPs, the average GRAVY value was found to be −0.53, and it was 0.1 for the QSPs. This difference was statistically significant and suggested that the ABPs are generally hydrophilic (negative GRAVY value) but the QSPs are mostly hydrophobic (positive GRAVY value).The GRAVY values of individual peptides from both datasets have been provided in Supplementary File [C].

#### Models based on the whole amino acid composition (Whole AAC)

**Figure 3:**
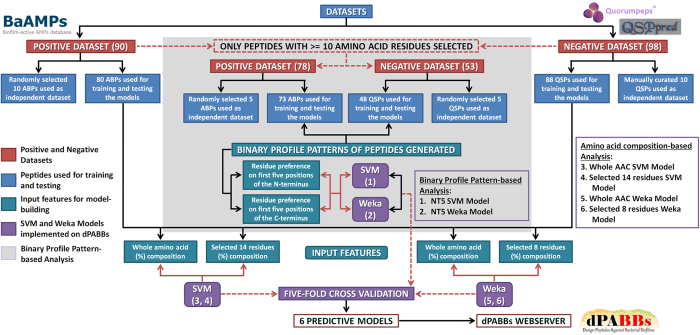
Flowchart depicting the overall approach implemented as the web server dPABBs. The flowchart gives an overview of the steps followed in building the predictive SVM and Weka-based models. These have been made functional on the web-based application dPABBs for predicting peptides with anti-biofilm activity.

Based on the observation that the ABPs and QSPs could be distinguished on the basis of their amino acid composition, models were generated with the whole amino acid (%) composition as the input feature (Fig. 3). The SVM Whole AAC based model achieved a maximum accuracy of 95.24% with sensitivity, specificity and MCC of 92.50, 97.73 and 0.91, respectively. Weka Whole AAC based model achieved a maximum accuracy of 95.24% with sensitivity, specificity and MCC of 93.75, 96.59 and 0.90, respectively. Thus, both SVM and Weka-based models performed with a prediction accuracy of >95% on the training datasets (Table 1). The models were evaluated using a five-fold cross validation technique as per the procedure illustrated in the Methods section.

#### Models based on selected features

The most important amino acid residues to be used as input features were selected for building the SVM and Weka-based models (Fig. 3). The best performances were achieved by the SVM model with selected 14 input features (maximum accuracy of 91.67% with sensitivity, specificity and MCC of 88.75, 94.32 and 0.83, respectively) and the Weka model with selected eight input features (maximum accuracy of 94.64% with sensitivity, specificity and MCC of 93.75, 95.45 and 0.89, respectively) (Table 2). The SVM model built with eight input features and the Weka model built with 14 input features along with other predictive models that were developed are available on request.

#### Positional preference of residues (Binary profile patterns)

In an attempt to utilize the information about the frequency and the preferred order of the residues at the N- and C- termini, predictive models using the binary profile patterns of the ABPs and QSPs were developed (Fig. 3). The accuracies of the SVM and Weka-based models trained on the NT5 dataset were 90.91% and 92.56%, with MCC values of 0.81 and 0.85 respectively, while those trained on the CT5 dataset performed with accuracies of 87.6% and 88.43%, the corresponding MCC values being 0.75 and 0.76 respectively (Table 3). To get best prediction results, only those BPP models with MCC ≥ 0.8 were selected.

**Figure 4:**
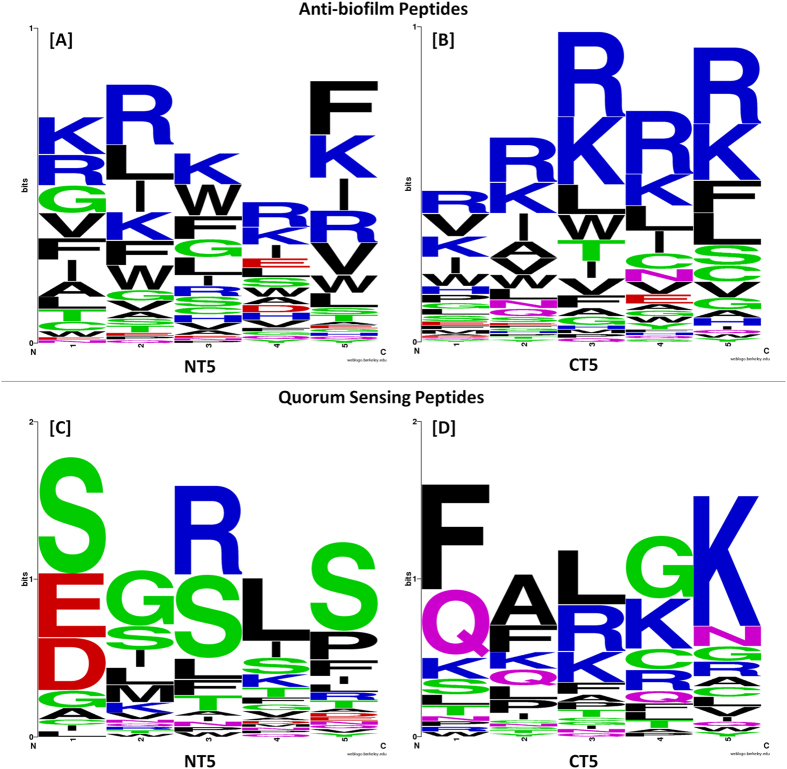
Sequence logos depicting amino acid residue preference at the first five positions of the N- and C-termini for ABPs and QSPs. (**A**) NT5 of ABPs (**B**) CT5 of ABPs (**C**) NT5 of QSPs (**D**) CT5 of QSPs.

In order to visualize the positional preference for specific amino acid residues in ABPs and QSPs, sequence logos were generated using the web application WebLogo^51^. Residue preference on the first five positions of the N- and C- termini (denoted by NT5 and CT5 respectively) is shown in Fig. 4. On the N-terminus, either one of the cationic polar amino acids, R and K, is present at all five positions in case of the ABPs, whereas in the QSPs, the uncharged polar amino acid S is preponderant at the first (followed by anionic polar amino acids D and E), third and fifth positions. On the C-terminus, while R and K are the most preferred amino acids at all five positions in the ABPs, in the QSPs, four out of the five amino acids most preferred at each position, namely F, A, L and G, are non-polar.

#### ROC plots

**Figure 5:**
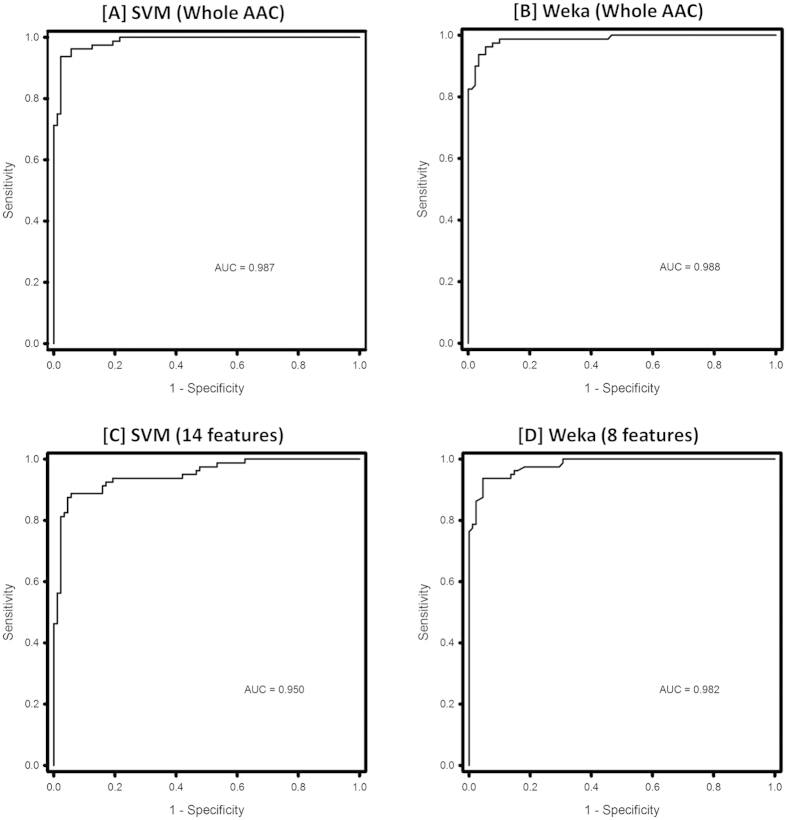
ROC curves for the models proposed in dPABBs. (**A**) SVM Whole AAC (**B**) Weka Whole AAC (**C**) SVM 14 features (**D**) Weka 8 features.

For a threshold-independent evaluation of the models, receiver operating characteristic (ROC) curves were plotted using SigmaPlot Version 11.0. The AUC provided a single value to gauge the performance of a classifier. The whole amino acid (%) composition based SVM (Fig. 5A) and Weka (Fig. 5B) models performed equally well but had marginally better AUC values when compared to the SVM model based on selected 14 features (Fig. 5C) and the Weka model based on selected 8 (Fig. 5D).

#### Performance on independent datasets

dPABBs proposes six models for predicting peptides with potential anti-biofilm activity. Positive and negative independent datasets of 10 peptides each were used to judge the predictive capacity of the four models based on amino acid composition, while datasets consisting of five peptides each were used to validate the two BPP models. The SVM model based on the whole amino acid composition of the peptides performed with the highest accuracy (with accuracy, sensitivity, specificity and MCC of 95%, 90%, 100% and 0.90, respectively) followed by the Weka model based on selected eight input features (amino acids, showed accuracy, sensitivity, specificity and MCC of 90%, 100%, 80% and 0.82, respectively). The SVM and Weka models trained on the NT5 dataset perform with an accuracy, sensitivity, specificity and MCC of 100%, 100%, 100% and 1 respectively, with the independent dataset of only five peptides (Table 4). Performances on both the training and independent datasets were considered to select the best models for the web server.

### Peptide drugs (FDA approved or in Clinical development) predicted to be anti-biofilm by dPABBs

A total of 242 sequences of biotech peptides/proteins were obtained from the DrugBank database. Of these, peptide sequences of length ≤36 amino acids were extracted and submitted on the MultiModel module of dPABBs to identify potential anti-biofilm activity. The prediction outcomes are displayed under the ‘Putative ABPs’ section ( http://ab-openlab.csir.res.in/abp/antibiofilm/feature.php) on the dPABBs web server. Out of the 31 peptides submitted, positive predictions were obtained for 29 FDA-approved peptide drugs. Only one peptide (DB04897) was predicted to be anti-biofilm by five out of the six models available on dPABBs (Supplementary File [D]).Corroborative analyses (BLAST sequence alignment, four base motif comparison and comparative assessment of the average amino acid residue % composition) suggest that these 31 FDA-approved peptide sequences were not direct derivatives of the 80 ABPs that constitute the positive dataset in this study (Supplementary File [D′]). Antimicrobial peptides in clinical trials or development were also analysed^28^. Positive predictions were obtained for all of the 10 peptides submitted. Some like Pexiganan acetate, Omiganan and hLF1-11 were predicted to be anti-biofilm by all the six models on dPABBs (Supplementary File [E]).

### Implementation and Utility of dPABBs

dPABBs is a user-friendly web server for predicting the anti-biofilm activity of a peptide and generating all possible mutants thereof to design potential ABPs with improved activity and optimised physicochemical properties. The server offers four different modules to the users, namely, Peptide, Protein, Batch and MultiModel. The Peptide module may be used for predicting the anti-biofilm activity of a single peptide and to generate its analogues with successive amino acid substitutions at each position. This feature helps the user select those mutants of the parent peptide which may have a higher SVM score/Weka probability and/or better physicochemical properties. The protein module generates all possible overlapping peptide fragments (with user-specified peptide length) and thus, helps in the identification of putative anti-biofilm peptides within a protein sequence. The Batch module is specially designed to screen putative ABPs from a library of peptides in a single run. The MultiModel module permits the selection of multiple models simultaneously for prediction. The user can then identify peptides which have been predicted to be biofilm-active by the maximum number of models and could be prioritized for testing. The results displayed in all the four modules (like the SVM score, Weka probability, desired physicochemical properties) can be sorted to view the values in ascending or descending order.

The dPABBs web server is accessible from the URL: http://ab-openlab.csir.res.in/abp/antibiofilm/.

## Discussion

A majority of the ABPs (57 out of the 90 peptides used in the study) were ≤18 residues in length, possibly because the shorter derivatives of known AMPs or minimally long synthetic peptides are generally desired and evaluated for retained or improved anti-biofilm activity^20^,^21^,^52^, more stability and cheaper production^53^, as well as reduced toxicity^52^. While QSPs are synthesised as longer pre-proteins, the processed signalling peptides are shorter fragments thereof, perhaps due to the high bioenergetic expense involved^54^. Hence, a majority of the QSPs (52 out of the 98 peptides used) were found to be ≤15 residues, with 18 of these being only 5 residues long. As a result of such a length distribution, peptides <10 residues long had to be excluded in order to carry out a BPP analysis of the ABPs and QSPs, such that 73 ABPs and 48 QSPs remained in the terminus datasets containing 5 N- and C-terminal residues of each peptide.

Abundant in cationic residues like K, H, R and polar residues like D, E, R, K, Q and N, antimicrobial peptides found to be active against biofilms (ABPs) possess a greater net charge than QSPs (with more anionic and neutral residues). An average GRAVY value of −0.53 indicates at their hydrophilic nature. These physicochemical properties could enable the ABPs to navigate the primarily aqueous environment of the EPS matrix. The EPS matrix (net negative charge) has been shown to sequester the positively charged antibiotic tobramycin (via ionic interaction with unknown matrix component), inhibit the penetration of negatively charged aminoglycosides, but allow ciprofloxacin, a neutral antibiotic to diffuse readily through the biofilm^55^,^56^. As noted before, the EPS constitutes ±85% of the biofilm, but the matrix itself is composed of ~95% water. This implies that a diffusing molecule should be hindered only when it directly interacts with an anionic component of the matrix, or when it is targeted specifically by the resident bacteria. In fact, it has been suggested that the net charged character of lactoferrin (a host-derived AMP) just marginally impedes its otherwise easy diffusion through *P. aeruginosa* biofilms^56^. At this juncture, the exact mechanisms are yet to be fully described, though steady advances are being made in the direction^57^.

Once formed, biofilms are notoriously tenacious. However, evidence for the adaptive nature of this recalcitrance comes from the demonstration that upon dispersal from a biofilm, the susceptibility to antibiotics is restored in the bacteria^58^. Therefore, while conventional resistance mechanisms like upregulated MDR efflux pumps, modifying enzymes, target mutations and mobile genetic resistance elements do contribute to biofilm persistence, the ABR associated with the residents of these complex structures can be more appropriately attributed to multicellular strategies^59^. These include (1) the EPS matrix delaying permeation of antibiotics (exposure to sub-inhibitory concentrations induces antibiotic stress in bacteria), (2) the antibiotic-degrading enzymes (like β-lactamases) accumulated in the matrix rendering these antibiotics inactive, (3) various gradients of nutrients, oxygen, and waste products leading to a heterogeneous population (differential states of growth and metabolism) not vulnerable to any single antibiotic, and (4) the presence of a persister subpopulation within the biofilm community^60^. This persister population relies on actively growing cells for the propagation of the genome whereas the latter depend on the former to re-establish the colony if the biofilm is eradicated^60^. Given the crucial role that the EPS matrix plays in recalcitrance, how its components interact with the antimicrobial agent determines the agent’s efficacy^56^.

In an attempt to identify possible drug repurposing candidates for anti-biofilm therapy, predictions were obtained for FDA-approved peptide drugs and AMPs in clinical development. Out of the 31 FDA-approved peptide drugs analysed, 29 were predicted as ‘biofilm-active’ by at least one and at most five models. An interesting observation was that the SVM and Weka NT5 BPP models gave positive predictions to the 10 peptides which were ‘biofilm-inactive’ according to the other four models that are based solely on the amino acid composition and do not factor in the positional preference of residues. Sinapultide (KL_4_), a 21 residue-long peptide designed to mimic the C-terminus of the human lung surfactant protein-B (SP-B), was the only FDA-approved peptide sequence to be predicted ‘biofilm-active’ by 5 out of the 6 models. Possessing particularly strong surface tension reducing properties, it forms a variable amphipathic helix in a lipid environment^61^. Lucinactant (DB04897), a gel-like suspension/liquid formulation for intratracheal use, contains sinapultide, the phospholipids POPG and DPPC along with a fatty acid (hexadecanoic acid, known as Palmitic acid) and is used for the treatment and prevention of respiratory distress syndrome (RDS) in premature infants. Lucinactant has also been approved as an orphan drug for cystic fibrosis; it has been found to promote mucus clearance^62^, and may perhaps also be effective against the bacterial biofilms infecting the lungs of cystic fibrosis patients. Sinapultide’s physicochemical resemblance to phenol soluble modulins (PSMs)^63^ is fascinating, especially when viewed in light of the prediction results obtained for the 13 Staphylococcal PSMs^64^ evaluated: 11 out of the 13 peptides were predicted as ‘biofim-active’ by at least two and at most six models of dPABBs.

Out of the 10 AMPs in clinical development that were evaluated for anti-biofilm activity, 6 peptides were predicted as ‘biofilm-active’ by all the six models. These include Omiganan (MX-226/MBI-226), OP-145 and Pexiganan acetate (MSI 78), which have been shown to be effective against catheter infections, chronic bacterial middle ear infection, and diabetic foot ulcers, respectively; biofilm involvement has been reported in all of these conditions^60^. These peptides could in fact serve as a bona fide validation set for the SVM and Weka-based models that have been made available on dPABBs.

To understand the limitations of these models, predictions were also obtained for the 26 QSPs with anti-biofilm/antimicrobial activity which had been excluded during the negative dataset preparation. The SVM Whole AAC model predicted the anti-biofilm activity of only six out of the 26 peptides, while the SVM model based on 14 selected residue features gave positive predictions for just two peptides. In comparison, the Weka-based models fared better, with the Weka Whole AAC model correctly predicting 12 out of 26 and the Weka model based on eight selected features predicting 14 out of the 26 (53.84%) peptides as being biofilm-active. The two BPP models, however, outperformed the remaining four in this regard. The SVM NT5 model could correctly predict the anti-biofilm activity of 20 out of the 26 peptides, but the Weka NT5 model could correctly classify 23 out of the 26 peptides (88.46%), achieving the best performance in terms of the percentage of peptides correctly predicted (Supplementary file [F]).

Research into the biofilm phenotype, spanning over almost three decades, has presented us with overwhelming evidence of its clinical relevance. Still, no approved drugs specifically targeting bacterial biofilms exist, and susceptibility testing against biofilms is conspicuously missing from antibiotic discovery pipelines. AMPs with activity against biofilms offer a promising avenue for the development of new standalone therapeutics or adjuvants acting synergistically with pre-existing antibiotics. However, since biofilms are perplexingly heterogeneous and dynamic, any predictions regarding the activity of these peptides should ideally be made in a species-specific manner, taking into account the stage of the biofilm lifecycle to be targeted. Going forward, as more peptides are experimentally evaluated for their activity against various bacterial biofilms, sufficient volumes of data would become available for building models capable of predictions with such specificity.

The dPABBs web server is an attempt to develop a prediction strategy for the identification and optimisation of such anti-biofilm peptides, offering a comprehensive platform that allows the user to check both peptides and protein fragments for potential anti-biofilm activity and provides features like simultaneous multi-model predictions and mutant generation.

## Methods

### Positive dataset (Anti-biofilm peptides extracted from BaAMPs database)

Initially, a total of 767 entries were retrieved from the BaAMPs database on 30 April 2015, out of which 652 were active and the remaining 115 were inactive peptides. After removing the duplicate peptide sequences (case sensitive removal), 140 unique active and 51 unique inactive peptides were obtained. VENNY^65^ was then used to identify and eliminate 33 peptides with inconclusive activity (peptides that were active against one species but inactive against the other). This elimination strategy yielded 107 exclusively active and 18 exclusively inactive peptides (Fig. 1A). The 107 exclusively active peptides were manually screened for structural modifications, properties or activity. In the process, 17 peptides that were cyclic, D-enantiomeric, coated, disulphide bond-containing, C-terminal spermidine conjugated, RNAIII inhibiting (QS) or anti-fungal were excluded to yield the final 90 peptide sequences (64 non-amidated linear +26 amidated linear) which were used to train and test the predictive models.

### Negative dataset (Quorum sensing peptides taken from QSPpred and cross referenced with Quorumpeps database)

The 18 inactive peptides obtained from the BaAMPs database could not constitute a substantive negative dataset and therefore quorum-sensing peptides (QSPs) were selected for this purpose. QSPs are oligopeptides used as autoinducing molecules by Gram positive bacteria in intra-species QS known to enable the biofilm phenotype. A total of 220 unique QSPs were retrieved from QSPpred web server comprising of positive (training + independent) datasets, from which one pyrrolysine (O) containing peptide sequence was removed. The remaining 219 peptides were then manually screened and simultaneously cross-referenced with the Quorumpeps database for corresponding entries. Entries were missing for 30 peptides in Quorumpeps, 68 peptides had cyclic structures and 7 amidated linear peptides were RNAIII inhibiting (RIPs) (Fig. 1B). Out of the remaining 114 non-amidated linear peptides, 26 were identified to have antimicrobial or biofilm-inhibitory activity (EDFs, RIPs, RBPs, bacteriocin inducing) and were excluded to yield the final 88 peptide sequences which were used to train the models. Additionally, 10 QSPs were retrieved from QSPpred (with missing Quorumpeps entries) and curated from published literature as the negative independent dataset.

### Support Vector Machine (SVM) and Weka

SVM-based classification was carried out by the means of the SVM^*light*^ version 6.02 software^66^, which has been used several times in the past to classify peptides based on their biological activity^32^,^33^. This study utilizes the Radial Basis Function (RBF) kernel from the SVM^*light*^ software. In addition to SVM^*light*^, we have also used Weka version 3.6.12, a Java based software package with various machine learning and feature selection algorithms^67^. Although multiple models were developed employing all the available modules in Weka, those based on the Random Forest (RF) and J48graft classifier were found to outperform the rest.

### Model Generation and Feature selection

In the past, studies have proven the importance of the amino acid composition, dipeptide and tripeptide composition percentage of peptide sequences in the development of classification models that predict their therapeutic potential^32^,^33^,^68^. Using these very parameters as the input features for SVM^*light*^ and Weka, 2500 SVM and 433 Weka-based models were build. The models performing with an accuracy of at least 85% on the independent dataset were selected. Of these, the best results were obtained using the amino acid composition as the input feature. Hence, the amino acid composition percentage was taken as the input feature for building the SVM and Weka models that were used on the web server. Amino acid composition percentage was calculated using the following formula:


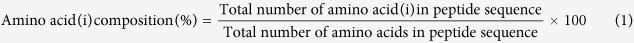


where i is any naturally occurring amino acid.

### Feature selection through Weka:

It has often been demonstrated that not all properties equally contribute to the biological activity of therapeutic peptides^69^. Thus, feature selection techniques may help in the identification of the most significant characteristics of the peptide that contribute to its anti-biofilm activity. Therefore, CfsSubsetEval attribute evaluator with BestFirst (with parameters: -D 1 -N 5) search method (using full training set as attribute selection mode), implemented in Weka is used to extract best features from amino acid, dipeptide and tripeptide composition percentage of peptides.

### Terminus datasets

In order to understand the role of N- and C-terminal residues in the ABPs and QSPs, terminus datasets were created using the N- and C-terminal residues of those peptides from main dataset which were ≥10 residues long. The following were derived from main dataset: (1) **NT5** contained first five residues (5 N-terminal residues) of 78 peptides; (2) **CT5** contained last five residues (5 C-terminal residues) of 53 peptides.

### Sequence logos

In order to determine the frequency with which various amino acids appeared at different positions in both the ABPs and QSPs, sequence logos were created using WebLogo software. The size of a residue’s logo represents its frequency at a given position. The height of the residue’s logo is also a measure of the residue variability at that position, in that the taller the logo, the lesser the variability at that position.

### Binary profile patterns (BPPs)

BPPs were generated for each peptide, where a vector of 20 dimensions represents each amino acid (*e.g.* Ala is represented by 1,0,0,0,0,0,0,0,0,0,0,0,0,0,0,0,0,0,0,0). A pattern of window length W was represented by a vector of dimensions 20× W. Binary profile patterns for the first 5 N-terminal residues and for the last 5 C-terminal residues were created for those peptides with ≥10 amino acid residues from the main datasets. In the N-terminal approach, five residues of the N-terminus were extracted from each peptide (NT5 subset), whereas in the C-terminal approach, five residues of the C-terminus were extracted from each peptide (CT5 subset). A binary profile of 5 × 20 dimensions was generated. These profiles were then used to develop SVM and Weka-based models. The BPP analysis has previously been used effectively^32^.

### Cross-validation technique

The performance of the models was evaluated by employing a five-fold cross-validation technique. The whole dataset was divided into five sets such that in each round, four sets were used for training and one was set aside for testing. Repeated five times, this ensured that each set was used once for testing the model that was trained on the remaining four.

### Performance measures

Once the classification models were ready, their performance was tested in terms of the accuracy, sensitivity, specificity and Mathew’s Correlation Coefficient (MCC). The following formulae were used for their calculation:


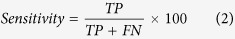



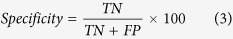



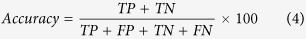



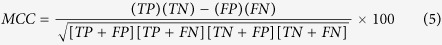


where TP and TN are correctly predicted positive and negative examples, respectively. Similarly, FP and FN are wrongly predicted positive and negative examples, respectively.

The models were also evaluated in a threshold independent manner by their receiver operating characteristic (ROC) curves and the areas under these curves (AUC). SigmaPlot 11.0 was used for generating the ROC curves and the calculating the corresponding AUC values.

### Independent datasets

To prepare the positive independent dataset, 10 anti-biofilm peptides were randomly selected from the final 90 anti-biofilm peptides. An equal number of QSPs were manually curated from published literature and QSPpred (peptides with missing Quorumpeps entries) for the negative independent dataset.

### Mutant Peptide Utility

In-house PERL scripts were written to generate mutants of the query peptides based on the user’s requirement. This feature was incorporated in dPABBs because substitutions of single amino acid residues have been shown to improve the anti-biofilm activity of biofilm-active peptides^18^,^21^. All the possible mutants are generated for the query peptide in such a way that only a single amino acid is substituted per cycle of mutant generation.

## Additional Information

**How to cite this article**: Gupta, P. *et al.* dPABBs: A Novel *in silico* Approach for Predicting and Designing Anti-biofilm Peptides. *Sci. Rep.* **6**, 21839; doi: 10.1038/srep21839 (2016).

## Supplementary Material

Supplementary Information

## Figures and Tables

**Table 1:** Performance of the best models based on the whole amino acid composition of the peptides.

Classifier	Sensitivity	Specificity	Accuracy	MCC	AUC
SVM	92.50	97.73	95.24	0.91	0.988
Weka	93.75	96.59	95.24	0.90	0.988

**Table 2:** Performance of the best models based on selected features (amino acid residues).

Classifier (No. of input features)	Sensitivity	Specificity	Accuracy	MCC	AUC
SVM (14)	88.75	94.32	91.67	0.83	0.953
Weka (8)	93.75	95.45	94.64	0.89	0.981

**Table 3:** Performance of the SVM and Weka models based on NT5 and CT5 datasets.

Classifier	Sensitivity	Specificity	Accuracy	MCC	AUC
SVM (NT5)	95.89	83.33	90.91	0.81	0.93
SVM (CT5)	97.26	72.92	87.6	0.75	0.92
Weka (NT5)	98.63	83.33	92.56	0.85	0.886
Weka (CT5)	90.41	85.42	88.43	0.76	0.879

**Table 4:** Performance of the proposed six models on independent datasets.

Classifier (No. of input features)	Sensitivity	Specificity	Accuracy	MCC
SVM (20)	90	100	95	0.90
SVM (14)	80	90	85	0.70
SVM (NT5)	100	100	100	1
Weka (20)	90	80	85	0.70
Weka (8)	100	80	90	0.82
Weka (NT5)	100	100	100	1
