# Supplementary material for: dPABBs: A Novel in silico Approach for Predicting and Designing Anti-biofilm Peptides
Source: Sci Rep. 2016 Feb 25;6:21839. doi: 10.1038/srep21839 (PMC4766436; doi:10.1038/srep21839)
Supplement: Supplementary Information [file srep21839-s1.pdf]

dPABBs: A Novel *in silico* Approach for Predicting and Designing Anti-biofilm Peptides

Pooja Gupta[1], Rakesh Kumar[1#], Arun Sharma[1#*], Anshu Bhardwaj[1#*]

[1]Open Source Drug Discovery (OSDD) Unit, Council of Scientific and Industrial Research, New Delhi, India.

[#]Academy of Scientific and Innovative Research, Delhi, India.

[*]bioinfo.arun@gmail.com
[*]anshub@osdd.net

*To whom the correspondence should be addressed:

Anshu Bhardwaj
Scientist, Open Source Drug Discovery (OSDD) Unit
Faculty Coordinator & Assistant Professor,
The Academy of Scientific & Innovative Research (AcSIR)
Council of Scientific and Industrial Research (CSIR)
Anusandhan Bhawan, 2 Rafi Marg, Delhi, India
+ 91 011 23470121
Skype: anshu.bhardwaj
http://ab-openlab.csir.res.in/

[A] List of the ABPs tested against the biofilms of Gram positive and Gram negative bacteria

S. No.	GRAM STATUS	BACTERIA	NUMBER OF ACTIVE PEPTIDES
1	Gram negative	*Acinetobacter baumannii*	3
2	Gram negative	*Burkholderia ambifaria*	1
3	Gram negative	*Burkholderia cenocepacia*	4
4	Gram negative	*Burkholderia dolosa*	1
5	Gram negative	*Burkholderia multivorans*	1
6	Gram negative	*Burkholderia pseudomallei*	3
7	Gram negative	*Burkholderia stabilis*	1
8	Gram negative	*Escherichia coli*	2
9	Gram negative	*Escherichia coli ATCC 25922*	4
10	Gram negative	*Escherichia coli HM22*	2
11	Gram negative	*Escherichia coli O-157 / ATCC 43895*	2
12	Gram negative	*Escherichia coli RP437*	2
13	Gram negative	*Klebsiella pneumoniae ATCC 13883*	1
14	Gram negative	*Porphyromonas gingivalis ATCC 33277*	1
15	Gram negative	*Pseudomonas aeruginosa*	18
16	Gram negative	*Pseudomonas aeruginosa ATCC 15442*	1
17	Gram negative	*Pseudomonas aeruginosa ATCC 27853*	10
18	Gram negative	*Pseudomonas aeruginosa PA14*	7
19	Gram negative	*Pseudomonas aeruginosa PAO1 / ATCC 15692*	13
20	Gram negative	*Salmonella enterica Typhimurium ATCC14028*	1
21	Gram positive	*Bacillus subtilis*	1
22	Gram positive	*Enterococcus faecalis*	2
23	Gram positive	*Enterococcus faecium ATCC 19434*	2
24	Gram positive	*Lactobacillus salivarius*	1
25	Gram positive	*Listeria iavanovii Li4pVS2*	1
26	Gram positive	*Propionibacterium acnes ATCC 6919*	1
27	Gram positive	*Staphylococcus aureus*	11
28	Gram positive	*Staphylococcus aureus ATCC 25923*	4
29	Gram positive	*Staphylococcus aureus ATCC 29213*	2
30	Gram positive	*Staphylococcus aureus ATCC 29737*	2
31	Gram positive	*Staphylococcus aureus ATCC 43300*	2
32	Gram positive	*Staphylococcus aureus ATCC 6538*	1
33	Gram positive	*Staphylococcus aureus Smith diffuse*	2
34	Gram positive	*Staphylococcus epidermidis ATCC 35984*	2
35	Gram positive	*Streptococcus gordonii*	1
36	Gram positive	*Streptococcus mutans*	4
37	Gram positive	*Streptococcus mutans ATCC 25175*	1

38	Gram positive	*Streptococcus mutans Clarke UA159 / ATCC 700610*	9
39	Gram positive	*Streptococcus mutans KCTC 3065*	1
40	Gram positive	*Streptococcus oralis ATCC 10557*	1
41	Gram positive	*Streptococcus parasanguinis ATCC 903*	1
42	Gram positive	*Streptococcus salivarius*	1
43	Gram positive	*Streptococcus salivarius ATCC 7073*	1
44	Gram positive	*Streptococcus sanguinis*	1
45	Gram positive	*Streptococcus sanguinis ATCC 10556*	1
46	Gram positive	*Streptococcus sanguinis NY101*	1
47	Gram positive	*Streptococcus sobrinus ATCC 6715*	1

[B] Length distribution of the ABPs and QSPs (Frequency of peptides of a particular length)

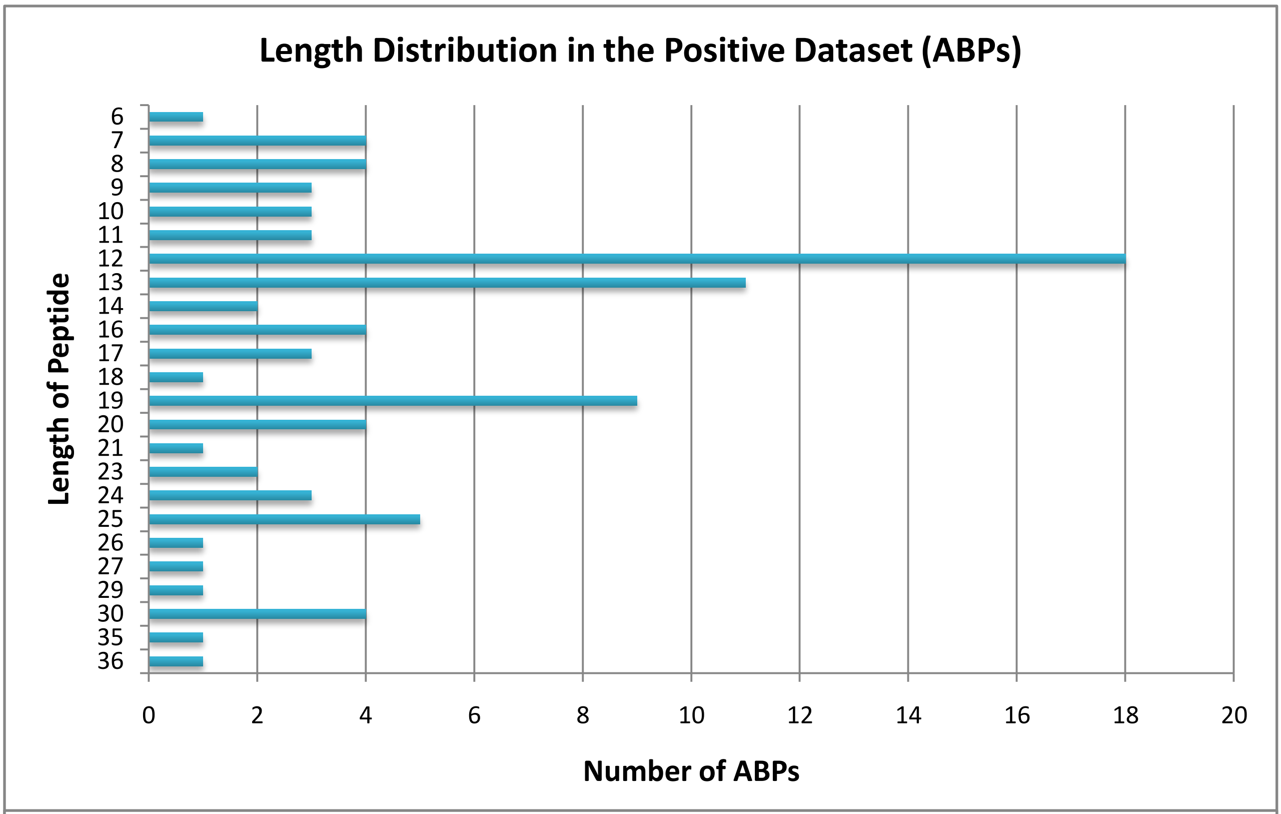

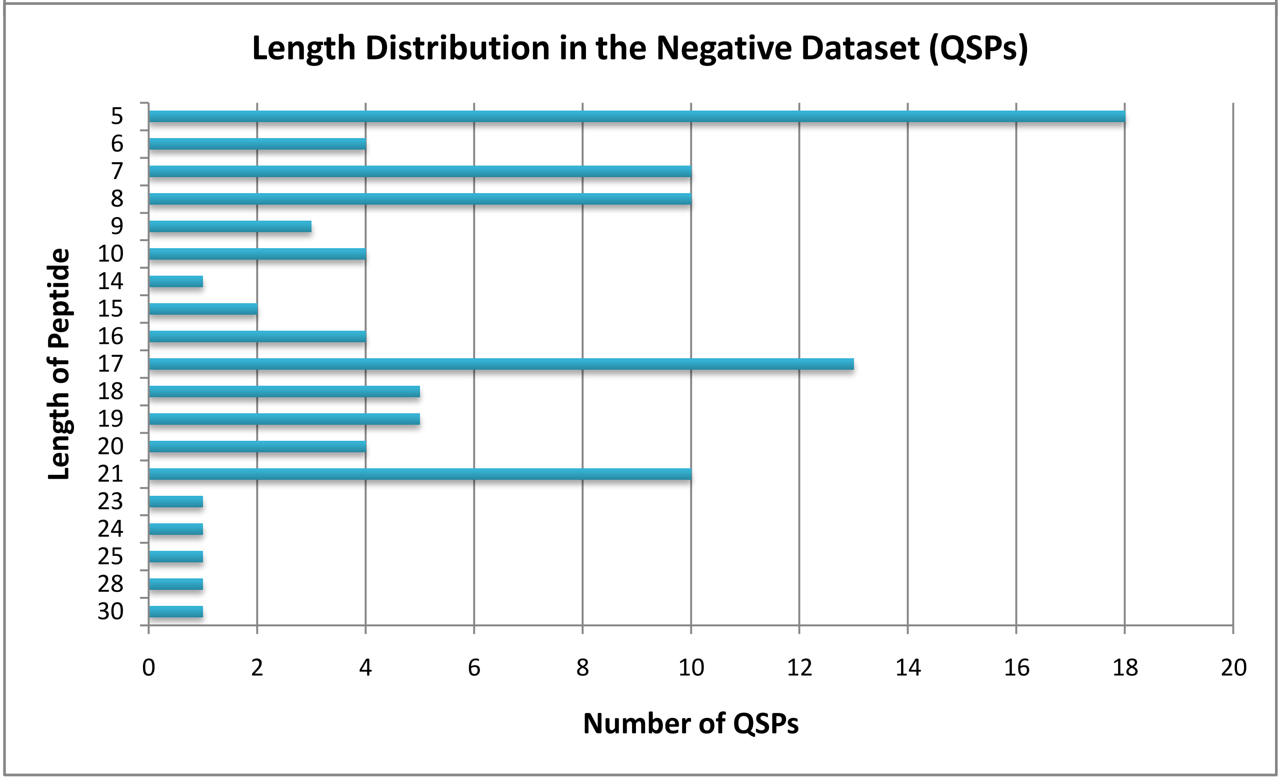

[C] GRAVY scores for ABPs and QSPs

ABPs (80)	GRAVY SCORE		QSPs (88)	GRAVY SCORE
FFGWLIKGAIHAGKAIHGLIHRRRH	0.00		AKDEH	-2.46
FIKHFIHRFGGGFKKFWKWFRRF	-0.43		AKTVQ	-0.42
FIKHFIHRFGGGRWRWRWF	-0.63		ARNQT	-2.08
FIKHFIHRFSATRWRWRWF	-0.55		DRVGA	-0.48
FKCRRWQWRMKKLG	-1.42		EKMIG	-0.28
FLGALFKALSKLL	1.48		ERGMT	-1.44
FLSLIPHIVSGVASIAKHF	1.26		ERNNT	-3.14
FRIRVRV	0.31		ERPVG	-1.16
GIFSKLAGKKIKNLLISGLKG	0.34		GKAEF	-0.64
GIGAVLKVLTTGLPALISWIKRKRQQ	0.27		LPFEF	0.86
GIGLFLHSAGLFGLAFVGEIMKS	1.22		LPFEH	-0.34
GKIIKLKASLKLL	0.71		MKAEH	-1.38
GLFDVIKKVASVIGGL	1.28		MPFEF	0.48
GRRRRSVQWCA	-1.37		QRGMI	-0.40
GRWKRWRKKWKKLWKKLS	-2.11		SKDYN	-2.60
GWGSFFKKAAHVGKHVGKAALTHYL	-0.07		SRNVT	-1.06
GWKKWLRKGAKHLGQAAIK	-0.85		VPFEF	0.94
HLGHHALDHLLK	-0.30		ADLPFEF	0.37
IGKEFKRIVERIKRFLRELVRPLR	-0.51		AITLIFI	3.03
IGKEFKRIVQRIK	-0.58		LVTLVFV	3.19
IGKEFKRIVQRIKDFLRNL	-0.46		NEVPFEF	-0.33
ILPWKWPWWPWRR	-1.07		SDLPFEH	-0.86
IRIKIRIK	0.15		SDMPFEF	-0.27
IRVKIRVKIRVK	0.08		SYPGWSW	-0.96
IRWRIRVWVRRIC	0.01		GLDWWSL	0.16
IVWKIKRWWVGR	-0.21		ALILTLVS	2.55
KIFGAIWPLALGALKNLIK	0.97		FLVMFLSG	2.26
KKHRKHRKHRKHGGSGGSKNLRRIIRKGIHI IKKYG	-1.68		LFSLVLAG	2.38
KQFRIRVRV	-0.58		LFVVTLVG	2.74
KRFRIRVRVIRK	-0.84		VAVLVLGA	2.93
KRIVQRIKDFLRNLVPRTES	-0.76		DIIIIVGG	2.24
KRWRIRVRVIRKC	-0.87		DILIIVGG	2.15
KRWRWIVRNIRR	-1.54		EGIIVIVVG	2.42
KRWWKWWRRC	-2.24		ILPYFAGCL	1.77
KSKEKIGKEFKRIVQRIKDFLRNLVPRTES	-1.05		CVFSLFKKCN	0.65
KWCFRVCYRGICYRKCR	-0.48		ILSGAPCIPW	1.18
KWFWKFVKFVK	-0.05		AILPYFAGCL	1.77
KWKLFKKIGIGKFLHSAKKF	-0.31		DLRNIFLKIKFKKK	-0.63
KWKVFKKIEKMGRNIRNGIVKAGPAIAVLG EAKAL	-0.07		DLRGVPNPWGWIFGR	-0.46

LAHQKPFIRKSYKCLHKRCR	-1.05		SNLVECVFSLFKKCN	0.45
LAREYKKIVEKLKRWLRQVLRTLR	-0.74		EMRKSNNNFFHFLRRI	-1.05
LLGDFFRKSKEKIGKEFKR	-1.06		DSRIRMGFDFSKLFGK	-0.48
LLGDFFRKSKEKIGKEFKRIVQRIK	-0.75		EIRQTHNIFFNFFKRR	-0.94
LRIRWIFKR	-0.30		EMRKPDGALFNLFRRR	-1.09
NEEGFFSARGHRPLDGGGKKKKKK	-1.78		DKRLPYFFKHLFSNRTK	-1.14
RFKRVARVIW	-0.08		DRRDPRGIIGIGKKLFG	-0.67
RFRRLFRIRVRVLKKI	-0.26		DWRISETIRNLIFPRRK	-0.96
RFWKVRVKYIRF	-0.42		EMRISRIILDFLFLRKK	0.19
RIVQRIKDFLRNLVPRTES	-0.59		EMRLPKILRDFIFPRKK	-0.66
RIWVIWRR	-0.26		EMRLSKFFRDFILQRKK	-0.83
RKSKEKIGKEFKRIVQRIK	-1.48		ESRLPKILLDFLFLRKK	-0.06
RKSKEKIGKEFKRIVQRIKDFLRNL	-1.17		ESRLPKIRFDFIFPRKK	-0.88
RKSYKALHKRAR	-1.93		ESRISDILLDFLFQRKK	-0.38
RKSYKCLHKRCR	-1.81		SGSLSTFFRLFNRSFTQ	-0.13
RPAFRKAAFRVMRACV	0.13		STFFRLFNRSFTQALGK	-0.16
RRWIRVAVILRV	0.69		GKATSSISKCVFSFFKKC	0.22
RRWVVWRIVQRR	-0.89		LSTFFRLFNRSFTQALGK	0.06
RWRWRW	-2.70		SGSLSTFFRLFNRSFTQA	-0.02
RWRWRWF	-1.91		SGTLSTFFRLFNRSFTQA	-0.02
RWRWRWRW	-2.70		SLSTFFRLFNFSFTQALG	0.64
TFFRLFNRGGGKNLRIIRKGIHIIKKY	-0.33		DIRHRINNSIWRDIFLKRK	-1.06
TFFRLFNRGGGWGSFFKKAAHVGKL	-0.10		GKPASNLVECVFSLFKKCN	0.14
TLISWIKNKRKQCRPRVSRRRRRRGGRRRR	-2.11		SGSLSTFFRLFNRSFTQAL	0.18
TLISWIKNKRKQRPRVSRRRRRRGGRRRR	-2.27		SLSTFFRLFNRSFTQALGK	0.02
TLISWIKNKRKQRPRVSRRRRRRGGRRRRC	-2.11		GSLSTFFRLFNRSFTQALGK	-0.01
VQFRIRVRIVIRK	0.27		SGSLSTFFRLFNRSFTQAGK	-0.24
VQWRIRVRVIKK	-0.34		SGSLSTFFRLFNRSFTQALG	0.15
VRLIRAVRAWRV	0.47		SGSLSTFFRLFNRSFTQALK	-0.03
VRLIVAVRIWRR	0.69		SGSLSTQFRLFNRSFTQALGK	-0.34
VRLIVRIWRR	0.23		SGSLSTFFLLFNRSFTQALGK	0.35
VRLIWAVRIWRR	0.27		SGSLSTFFRLFLRSFTQALGK	0.30
VRLRIRVA	0.63		SGSLSTFFRLFNASFTQALGK	0.26
VRLRIRWWVLRK	-0.27		SGSLSTFFRLFNRSFTQALGA	0.23
VRWARVARILRV	0.47		SGSLSTFFRLFNRSFTQALGK	-0.04
VTCDVLSFEAKGIAVNH	0.61		SGSLSTFFRLFNRSFTQALGV	0.34
WIVVIWRRKRRRC	-0.64		SGSLSTFFRLFNRSQTQALGK	-0.34
WKLLSKAQEKFGKNKSR	-1.48		SGSLSTFFRLQNRSFTQALGK	-0.34
WRWRVRVWR	-1.37		SQKGVYASQRSFVPSWFRKIFRN	-0.70
YAPWTNA	-0.63		AGTKPQGKPASSISKCVFSFFKKC	-0.22
YAPWTNF	-0.49		SINSQIGKATSNLVECVFSLFKKCN	0.16
**Average ABPs**	**-0.53**		EQLSFTSIGILQLLTIGTRSCWFFYCRY	0.49
			SGWMDYINGFLKGFGGQRTLPTKDY NIPQV	-0.53
			SRNAT	-1.54

			AIFILAS	2.63
			SIFTLVA	2.23
			EIIIIVGG	2.24
			ESRVSRIILDFLFQRKK	-0.41
			SGSLSTFFRLFNFSFTQALGK	0.30
			**Average QSPs**	**0.10**

For all other properties (such as amino acid percentage composition, physicochemical properties) similar analysis (two sample t-test assuming unequal variance, using Microsoft Office 2007) was performed to determine statistical significance (p-value < 0.05).

[D] Prediction on FDA approved biotech peptides and examples of active/inactive mutant peptides with improved SVM scores

DrugBank ID	Parent peptide	Predicted as Biofilm-active (No. of models)	Mutated peptide	Predicted as Biofilm-active (by SVM Whole AAC)	SVM score (Parent / Mutant)
DB04921	DENPVVHFFKNIVTPRT	Yes (1)	DENPVVHRFKNIVTPRT	Yes	0.46 / 0.92
DB01309	FVKQHLCGSHLVEALYLVCGERGFFYTPET	Yes (3)	FVKQHLCGSHLVEALYLVCGERGFFYTHET	Yes	0.31 / 0.71
DB01306	FVNQHLCGSHLVEALYLVCGERGFFYTDKT	Yes (3)	FVNQHLCGHHLVEALYLVCGERGFFYTDKT	Yes	0.33 / 0.72
DB00046	FVNQHLCGSHLVEALYLVCGERGFFYTKPT	Yes (3)	FVNQHLCGSHLVEALYLVCGERGFFYTKHT	Yes	0.30 / 0.71
DB00030; DB00071; DB08914	FVNQHLCGSHLVEALYLVCGERGFFYTPKT	Yes (3)	FVNQHLCGSHLVEALYLVCGERGFFYTHKT	Yes	0.30 / 0.71
DB00047	FVNQHLCGSHLVEALYLVCGERGFFYTPKTRR	Yes (3)	FVNQHLCGKHLVEALYLVCGERGFFYTPKTRR	Yes	0.56 / 0.96
DB05875	RPKPQQFFGLM	Yes (3)	RHKPQQFFGLM	No	-1.31 / -0.54
DB08888	APSFDCGKPQVEPKKCPGR	Yes (4)	AHSFDCGKPQVEPKKCPGR	No	-1.12 / -0.56
DB01307	FVNQHLCGSHLVEALYLVCGERGFFYTPK	Yes (4)	FVNQHLCGSHLVEALYLVCGERGFFYTHK	Yes	0.29 / 0.73
DB06655	HAEGTFTSDVSSYLEGQAAKEEFIIAWLVKGRG	Yes (4)	HAEGTFTSHVSSYLEGQAAKEEFIIAWLVKGRG	Yes	0.42 / 0.83
DB05332	IEGPTLRQWLAARA	Yes (4)	IEGPTLRQWLVARA	Yes	0.13 / 0.79
DB05685	MKHLLLLLLCVFLVKSQGVNDNEEGFFS	Yes (4)	MKHLLLLLLCVFLVKHQGVNDNEEGFFS	Yes	-0.37 / 0.10
DB04897	KLLLLKLLLLKLLLLKLLLLK	Yes (5)	KALLLKLLLLKLLLLKLLLLK	No	-0.49 / -0.33
DB06285	SVSEIQLMHNLGKHLNSMERVEWLRKKLQDVHNF	Yes (3)	VVSEIQLMHNLGKHLNSMERVEWLRKKLQDVHNF	Yes	0.57 / 0.97
DB01284	SYSMEHFRWGKPVGKKRRPVKVYP	Yes (4)	SYSMEHFRWGKHVGKKRRPVKVYP	Yes	0.57 / 1.06
DB00017	CSNLSTCVLGKLSQELHKLQTYPRTNTGSGTP	Yes (2)	CHNLSTCVLGKLSQELHKLQTYPRTNTGSGTP	No	-0.69 / -0.30
DB00067	CYFQNCPRG	Yes (2)	CYFQNCRRG	No	-1.21 / -0.53
DB00107	CYIQNCPLG	Yes (2)	CYIQNCHLG	No	-1.24 / -0.47
DB00006	FPRPGGGGNGDFEEIPEEYL	Yes (2)	FHRPGGGGNGDFEEIPEEYL	No	-1.63 / -1.23
DB00047	GIVEQCCTSICSLYQLENYCG	Yes (2)	GIVEQCCTHICSLYQLENYCG	No	-0.89 / -0.43

DrugBank ID	Parent peptide	Predicted as Biofilm-active (No. of models)	Mutated peptide	Predicted as Biofilm-active (by SVM Whole AAC)	SVM score (Parent / Mutant)
DB00030; DB00046; DB00071; DB01306; DB01307; DB01307; DB01309; DB08914	GIVEQCCTSICSLYQLENYCN	Yes (2)	GIVEQCCT**H**ICSLYQLENYCN	No	-0.95 / -0.48
DB08900	HGDGSFSDEMNTILDNLAARDFINWLIQTKITD	Yes (1)	HG**V**GSFSDEMNTILDNLAARDFINWLIQTKITD	No	-1.19 / -0.71
DB00021	HSDGTFTSELSRLRDSARLQRLLQGLV	Yes (1)	H**H**DGTFTSELSRLRDSARLQRLLQGLV	No	-0.93 / -0.40
DB00040	HSQGTFTSDYSKYLDSRRAQDFVQWLMNT	Yes (1)	H**H**QGTFTSDYSKYLDSRRAQDFVQWLMNT	No	-1.36 / -0.85
DB04985	PGDSTRKCMDLKGNK	Yes (1)	PG**H**STRKCMDLKGNK	No	-0.95 / -0.15
DB00007	PHWSYLLR	Yes (2)	PHW**K**YLLR	Yes	-0.45 / 0.39
DB04900	SDAAVDTSSEITTKDLKEKKEVVEEAEN	No	**V**DAAVDTSSEITTKDLKEKKEVVEEAEN	Yes	-0.20 / 0.08
DB04899	SPKMVQGSGCFGRKMDRISSSSGLGCKVLRRH	No	**H**PKMVQGSGCFGRKMDRISSSSGLGCKVLRRH	No	-0.62 / -0.13
DB05671	TDLQERGDNDISPFSGDGQPFKD	Yes (2)	TDLQERGDNDI**V**PFSGDGQPFKD	No	-2.20 / -1.78
DB00010	YADAIFTNSYRKVLGQLSARKLLQDIMSRQ	Yes (2)	YADAIFTN**H**YRKVLGQLSARKLLQDIMSRQ	No	-0.61 / -0.04
DB00109	YTSLIHSLIEESQNQQEKNEQELLELDKWASLWNWF	Yes (2)	YT**H**LIHSLIEESQNQQEKNEQELLELDKWASLWNWF	No	-0.61 / -0.30

[E] Prediction on some antimicrobial peptides in clinical development. The table lists the peptide name, peptide sequence, trial phase and prediction on the peptide

Peptide Name	Peptide Sequence	Clinical Stage (Phase)	Prediction (No. of models)
Pexiganan acetate (MSI 78)	GIGKFLKKAKKFGKAFVKILKK	III	Biofilm-active (6)
Omiganan (MX-226/MBI-226)	ILRWPWWPWRRK	III	Biofilm-active (6)
Omiganan (CLS001)	ILRWPWWPWRRK	II / III	Biofilm-active (6)
Iseganan (IB-367)	RGGLCYCRGRFCVCVGR	III	Biofilm-active (5)
hLF1-11	GRRRRSVQWCA	I / II	Biofilm-active (6)
PAC-113	AKRHHGYKRKFH	IIb	Biofilm-active (6)
CZEN-002	CKPVCKPV	IIb	Biofilm-active (3)
IMX942	KSRIVPAIPVSLL	Ia	Biofilm-active (3)
OP-145	IGKEFKRIVERIKRFLRELVRPLR	II (completed)	Biofilm-active (6)
Ghrelin	GSSFLSPEHQRVQQRKESKKPPAKLQPR	II	Biofilm-active (1)

[F] Analysis of 26 QSPs with anti-biofilm activity: percentage of correct predictions made by the 6 models on dPABBs

Models	Number of correct predictions	Total number of peptides	Percentage of correct predictions (%)
SVM (20)	6	26	23.07
SVM (14)	2	26	7.69
Weka (20)	12	26	46.15
Weka (8)	14	26	53.84
SVM (NT5)	20	26	76.92
Weka (NT5)	23	26	88.46

[D`] Analyses to ascertain whether the 31 FDA-approved peptide sequences are derivatives of the 80 anti-biofilm peptides (positive dataset used in training)

In the first analysis, a database of the anti-biofilm peptides (ABPs) was created and BLAST (blastp) was performed for each of the FDA-approved peptides against this database (e-value 0.0001, other default parameters). None of the FDA-approved peptides showed a match with any ABP. From this sequence alignment data, it can be concluded that the ABPs and the FDA-approved peptides do not share any significant sequence similarity.

In the second analysis, a library of four amino acid-long peptides was created from each FDA-approved peptide and ABPs, using a step size of one amino acid. This library obtained from FDA-approved peptides was then compared with that obtained from the ABPs for exactly matching four-amino acid sequences. Seven four-amino acid stretches (GQAA, NEEG, EEGF, EGFF, GFFS, LKLL, WLRK) were found to be common among these. They belong to the following four FDA-approved peptides from the 31 used in the study:

FOUR-RESIDUE MOTIF	FDA-APPROVED SEQUENCE	ANTI-BIOFILM PEPTIDE
GQAA	HAEGTFTSDVSSYLEGQAAKEEFIIAWLVKGRG	GWKKWLRKGAKHLGQAAIK
NEEG	MKHLLLLLLCVFLVKSQGVNDNEEGFFS	NEEGFFSARGHRPLDGGGKKKKKK
EEGF	MKHLLLLLLCVFLVKSQGVNDNEEGFFS	NEEGFFSARGHRPLDGGGKKKKKK
EGFF	MKHLLLLLLCVFLVKSQGVNDNEEGFFS	NEEGFFSARGHRPLDGGGKKKKKK
GFFS	MKHLLLLLLCVFLVKSQGVNDNEEGFFS	NEEGFFSARGHRPLDGGGKKKKKK
LKLL	KLLLLKLLLLKLLLLKLLLLK	GKIIKLKASLKLL
WLRK	SVSEIQLMHNLGKHLNSMERVEWLRKKLQDVHNF	GWKKWLRKGAKHLGQAAIK

Additionally, the average composition % for each of the amino acid residue was also compared for the two sets of peptides, as shown in the graphs below. It is thus evident that the FDA-approved peptides and the ABPs have different amino acids residue compositions.

Based on these three analyses, it can be safely concluded that the 31 FDA-approved peptides and the 80 ABPs have different sequences and compositions of amino acids, and related or derivatives of each other.

[A] Average Amino Acid Residue Composition % - ABPs versus FDA-approved Peptide Sequences

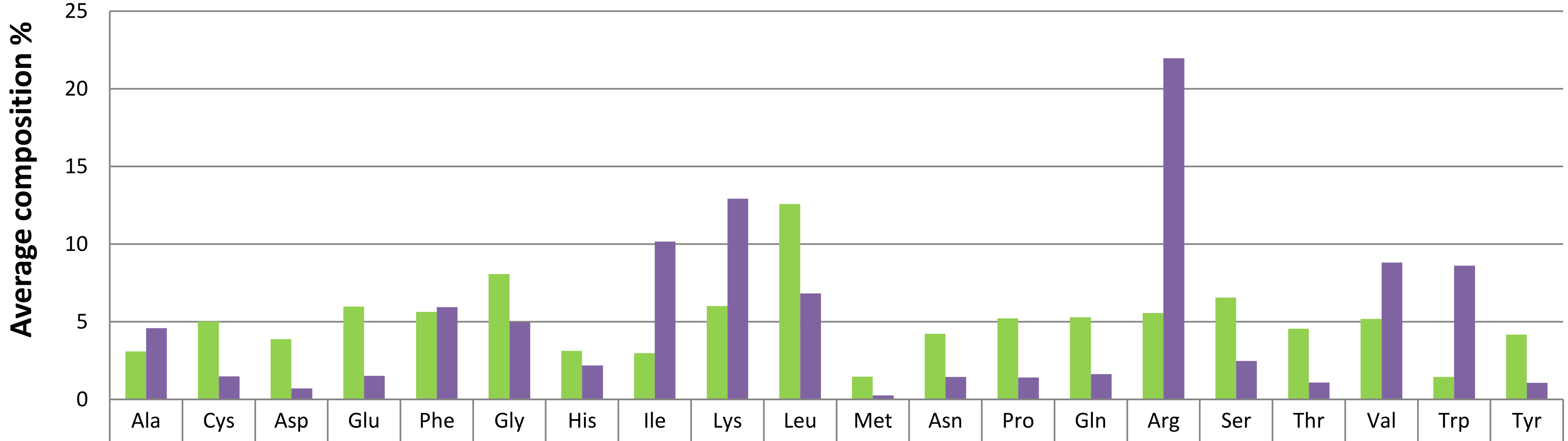

	Ala	Cys	Asp	Glu	Phe	Gly	His	Ile	Lys	Leu	Met	Asn	Pro	Gln	Arg	Ser	Thr	Val	Trp	Tyr
FDA-approved peptide drugs	3.078	5.02	3.88	5.969	5.63	8.069	3.129	2.986	6.004	12.574	1.466	4.221	5.215	5.297	5.551	6.555	4.554	5.188	1.438	4.175
Anti-biofilm peptides (ABPs)	4.586	1.479	0.709	1.516	5.943	4.965	2.182	10.156	12.923	6.818	0.257	1.447	1.402	1.62	21.965	2.464	1.087	8.804	8.613	1.062

[B] Fold-change in the Average Amino Acid Residue Composition % - ABPs versus FDA-approved Peptide Sequences

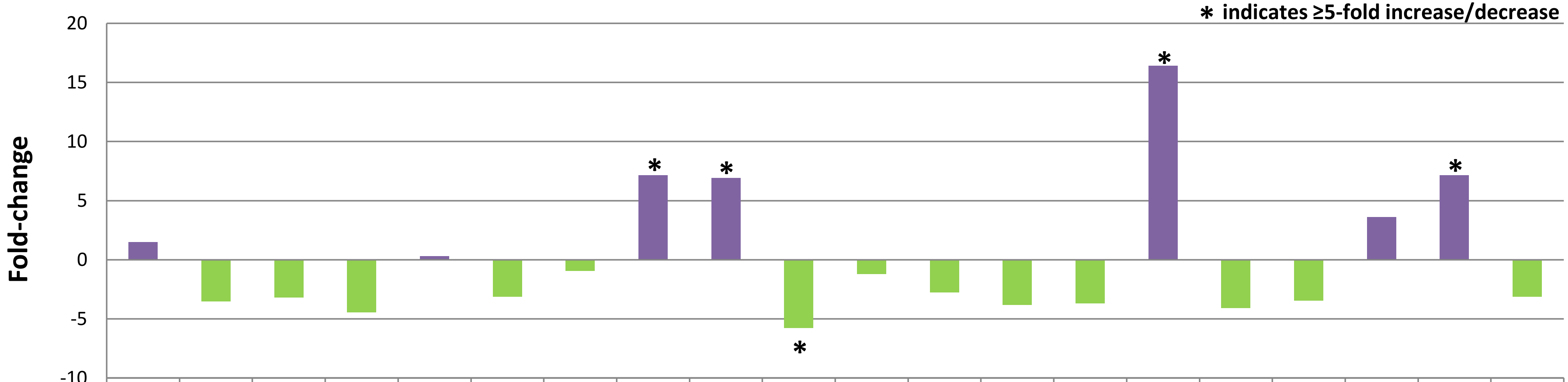

	Ala	Cys	Asp	Glu	Phe	Gly	His	Ile	Lys	Leu	Met	Asn	Pro	Gln	Arg	Ser	Thr	Val	Trp	Tyr
Fold-change	1.508	-3.541	-3.171	-4.453	0.313	-3.104	-0.947	7.17	6.919	-5.756	-1.209	-2.774	-3.813	-3.677	16.414	-4.091	-3.467	3.616	7.175	-3.113
